# Self-induced synthesis of phase-junction TiO_2_ with a tailored rutile to anatase ratio below phase transition temperature

**DOI:** 10.1038/srep20491

**Published:** 2016-02-11

**Authors:** Wei-Kang Wang, Jie-Jie Chen, Xing Zhang, Yu-Xi Huang, Wen-Wei Li, Han-Qing Yu

**Affiliations:** 1 CAS Key Laboratory of Urban Pollutant Conversion, Department of Chemistry, University of Science & Technology of China, Hefei, 230026, China

## Abstract

The surface phase junction of nanocrystalline TiO_2_ plays an essential role in governing its photocatalytic activity. Thus, facile and simple methods for preparing phase-junction TiO_2_ photocatalysts are highly desired. In this work, we show that phase-junction TiO_2_ is directly synthesized from Ti foil by using a simple calcination method with hydrothermal solution as the precursor below the phase transition temperature. Moreover, the ratio of rutile to anatase in the TiO_2_ samples could be readily tuned by changing the ratio of weight of Ti foil to HCl, which is used as the hydrothermal precursor, as confirmed by the X-ray diffraction analysis. In the photocatalytic reaction by the TiO_2_ nanocomposite, a synergistic effect between the two phases within a certain range of the ratio is clearly observed. The results suggest that an appropriate ratio of anatase to rutile in the TiO_2_ nanocomposite can create more efficient solid-solid interfaces upon calcination, thereby facilitating interparticle charge transfer in the photocatalysis.

Titania is an intriguing material with wide applications in the fields of water splitting^1^, solar cell^2^, pollutant degradation^3^,^4^, and lithium-ion batteries^5^. It has been recognized as one of the most promising and suitable photocatalytic materials for energy conversion and environmental remediation due to its abundant availability, nontoxicity, and high chemical stability. In principle, TiO_2_ is recognized as an ideal catalyst, but its capability to efficiently separate photoinduced electrons and holes needs substantial improvement^6^,^7^. Since photoinduced electrons and holes of anatase or rutile phase in TiO_2_ are easy to combine, resulting in their poor availability on the surface and corresponding limited photocatalytic activity. It is reported that crystal form might be the most significant factor governing the photocatalytic performance of TiO_2_^8^.

Today, it is widely recognized that the mixed phase of TiO_2_ is beneficial to reducing the recombination of photogenerated electrons and holes, which results in an enhanced photocatalytic activity. In particular, Degussa P25, a successful commercialized TiO_2_^9^, as a typical phase-mixed TiO_2_, has been extensively used in many fields^10^,^11^. Recently, the phase junctions of mixed-phase TiO_2_ have attracted considerable attention because they exhibit better photocatalytic performance than the pure single phase. Due to the difference in their conduction bands, the charge transfer from one phase to another makes it possible to prolong charge lifetime for photocatalytic reactions. Also, the robust separation of photoexcited charge carriers between the two phases and an efficient route to improve photocatalysts have been extensively reported and recognized^12^,^13^,^14^,^15^.

To date, several methods to synthesize such a phase-junction TiO_2_ have been reported. For instance, dose of organic reagents and anions (e.g., NO_3_^−^ and SO_4_^2−^) into the preparation precursor has been used to synthesize mixed-phase TiO_2_ nanoparticles^13^,^16^,^17^,^18^. TiO_2_ films containing the phases of anatase and rutile were prepared by advanced plasma electrolytic oxidation of pure titanium foils^14^. Surface anatase/rutile junction was prepared by calcinating the tianium isopropoxide hydrolyzed on commercial rutile particles^15^,^19^. Also, template methods have been used to achieve the phase transition by adjusting the calcination temperature. Although substantial progress has been made in preparing TiO_2_-based materials with a better photocatalytic activity^20^,^21^,^22^,^23^,^24^,^25^,^26^, development of more facile and simple methods for the synthesis of tunable phase-junction TiO_2_ is still highly desired.

In this work, a simple but effective calcination method was developed to synthesize phase-junction TiO_2_ nanocomposites below the phase transition temperature with a tunable anatase to rutile ratio using the hydrothermal solution containing Ti foil and hydrochloric acid without any adjustors as the precursor. Photocatalytic activities of the phase-junction TiO_2_ nanocomposites were evaluated in the degradation of Rhodamine B (RhB) and the hydrogen production tests. In this way, a novel strategy for self-controllable phase junction engineering of TiO_2_ was successfully developed.

## Results

### Structural and chemical characteristics of the phase-junction TiO_2_ nanocomposites

**Figure 1:**
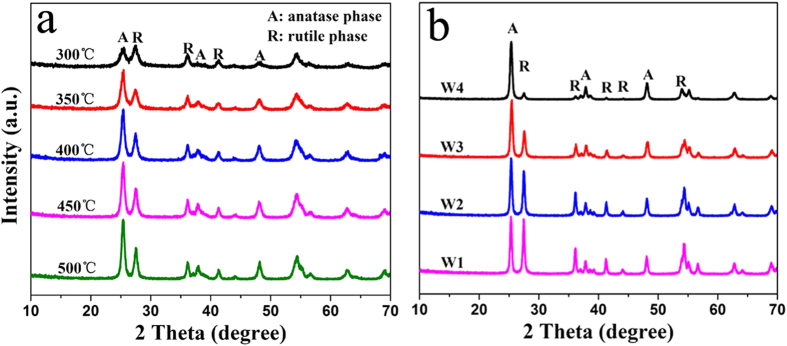
XRD patterns of the phase-junction TiO_2_ nanoparticles: (**a**) the crystallization process with the increasing annealing temperature; (**b**) obtained with 0.6 g of Ti (W1), 0.4 g of Ti (W2), 0.2 g of Ti (W3), and 0.1 g of Ti (W4).

Figure 1a shows the X-ray diffraction (XRD) patterns of the phase-junction TiO_2_ nancomposites prepared by annealing the precursor at different temperatures. The crystallinity of the samples increased with the increasing annealing temperature. Since the photocatalytic activity of TiO_2_ is dependent greatly on its crystallinity^27^, the crystallinity of the samples W1-W4 could be ensured by annealing at 500 °C (Fig. 1a). It should be noticed that this temperature did not change the phase of the obtained samples until 600 ^o^C (Supplementary Figure S1a).

The XRD patterns of the samples (Fig. 1b) exhibited similar diffraction peaks, indicating that the obtained samples were the TiO_2_ nanocomposites consisting of anatase and rutile nanoparticles. Figure 1 clearly shows the coexistence of two titania polymorphs, i.e., tetragonal rutile (JCPDS No. 21-1276) and tetragonal anatase (JCPDS No. 21-1272). The existence of rutile in the nanocomposites was readily discernible from its (110) diffraction peak located at 2 theta of 27.4° in the XRD pattern, because no overlapping of this peak with any other peaks from anatase occurred. Anatase phase can be also easily identified from its (101) peak located at 2 theta of 25.3°, as this peak doesn’t overlap with any other peaks of rutile. This result clearly demonstrates that rutile and anatase coexisted in the samples W1-4. However, the XRD pattern clearly shows that W5 exhibited anatase phase only (Supplementary Figure S1b).

The weight percentage of rutile phase can be calculated from the individual diffraction peaks using a previously reported method^18^. The phase contents of the samples can be estimated from the respective XRD peak intensities with the following equation:


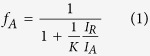



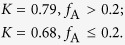


where *f*_A_ is the fraction of anatase phase in the powder, and *I*_A_ and *I*_R_ are the X-ray intensities of the anatase (101) and rutile (110) diffraction peak, respectively.

From the XRD patterns of W1-W4 (Fig. 1b), it is estimated that the weight percentages of rutile in the four mixed-phase nanocomposites were 51.8, 46.9, 34.4, and 11.6%, respectively. This result indicates that the rutile to anatase ratio of the nanocomposites could be tuned over a wide range with our synthesis approach. In addition, this ratio was not substantially affected by the hydrothermal time or the Ti precursor preparation temperature (Supplementary Figure S2a,b). Since HCl would react with Ti foil, the lesser dose of Ti foil would result in a higher HCl/Ti ratio^28^. Thus, the HCl/Ti ratio of the Ti solutions increased from W1 to W4, and consequently led to a decrease in rutile content for the obtained samples. Such a result is also consistent with previous reports that rutile formation was suppressed at high HCl/Ti ratios^28^.

The formation mechanism of the phase-junction TiO_2_ could be explained from both experimental result and first-principle calculations. The enhanced photocatalytic performance of the anatase/rutile nanocomposites over pure anatase or rutile nanocrystals might be contributed by the synergistic effects^29^ and the built-in electric field at the interface^30^,^31^,^32^. The possible mixed-phase junctions between rutile and anatase included: rutile (101)/anatase (001) with the closest bonding arrangements and parameters^30^; rutile (100)/anatase (100) with a nice fit rearrangement of the top anatase layer to form a rutile-like layer^33^, and rutile (111)/anatase (101) with well-matched lattice fringes^31^. The high-resolution transmission electron microscopy (HRTEM) analysis shows that our TiO_2_ samples with a tunable phase ratio might contain all the above possible phase junctions, and also include some junctions from crystal surface with relatively high energies, resulting in a stronger tendency to form interfaces^34^,^35^.

**Figure 2:**
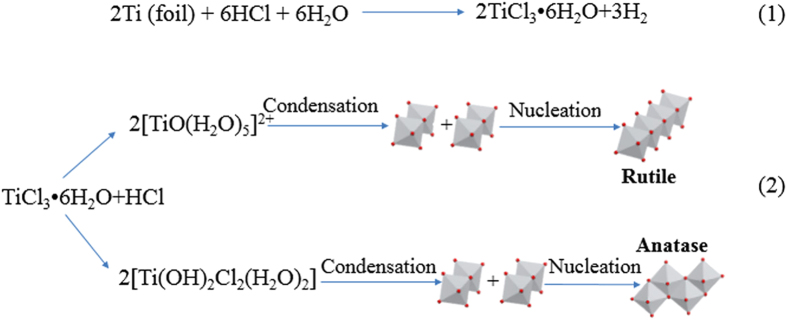
Proposed formation reactions of the phase-junction TiO_2_.

**Figure 3:**
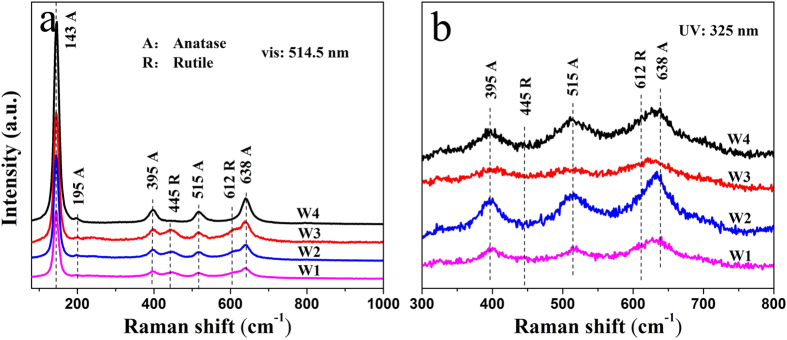
(**a**) Visible Raman spectra and (**b**) UV Raman spectra of the obtained TiO_2_ samples with the excitation lines at 514.5 nm and 325 nm respectively.

The formation reactions of the two-phase TiO_2_ are proposed as shown in Fig 2^36–38^. The Raman spectra of the samples in Fig. 3 also reveal similar information about the bulk TiO_2_ with XRD patterns^15^. From visible Raman spectra (Fig. 3a), two important phenomena were observed: 1) no obvious broadening of anatase E_g_ mode at 143 cm^−1^, suggesting that the crystal sizes of the samples were of similar levels; and 2) two rutile modes at 445 cm^−1^ and 612 cm^−1^ for W1, W2, and W3, except for W4, indicating that the rutile phase was almost absence in the bulk phase of W4^21^. Additionally, due to its high sensitivity for surface phase detection, UV Raman spectra could offer useful information about the surface phase structure of the samples. As shown in Fig. 3b, both anatase and rutile modes were observed for the samples W1-4, confirming that both anatase and rutile phases were present in the surface region. The above XRD patterns and visible and UV Raman spectra results show that the rutile phase of W4 was mainly located on the surface^39^.

**Figure 4:**
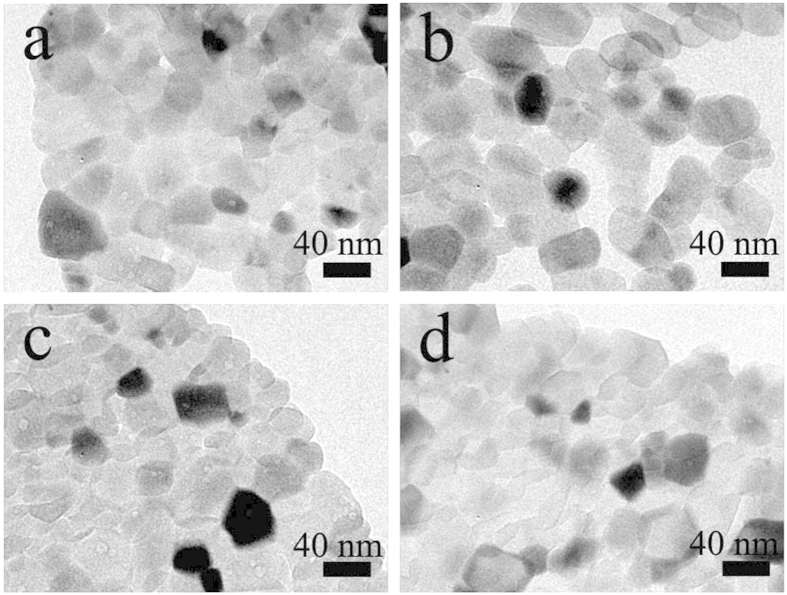
Typical TEM images of the phase-junction TiO_2_ sample W1 (**a**), W2 (**b**), W3 (**c**), and W4 (**d**).

The low-magnification TEM images of the phase-junction TiO_2_ samples synthesized from Ti^3+^ with different concentrations are shown in Fig. 4. All these samples showed similar morphologies, i.e., aggregates of TiO_2_ nanoparticles with a size of about 40 nm. This result is consistent with the visible Raman observation that all the samples had a similar crystal size. The TEM images of the particles after Pt deposition are shown in Supplementary Figure S3, indicating that Pt nanoparticles were uniform and had a diameter of approximately 2 nm in all the phase-junction samples. Furthermore, the ICP results show that the Pt content of the samples W1–4 were 0.54, 0.59, 0.53, and 0.55 μg/mL, respectively. Thus, the ICP results and TEM images demonstrate that the amounts of Pt were almost of the same level for all the samples.

**Figure 5:**
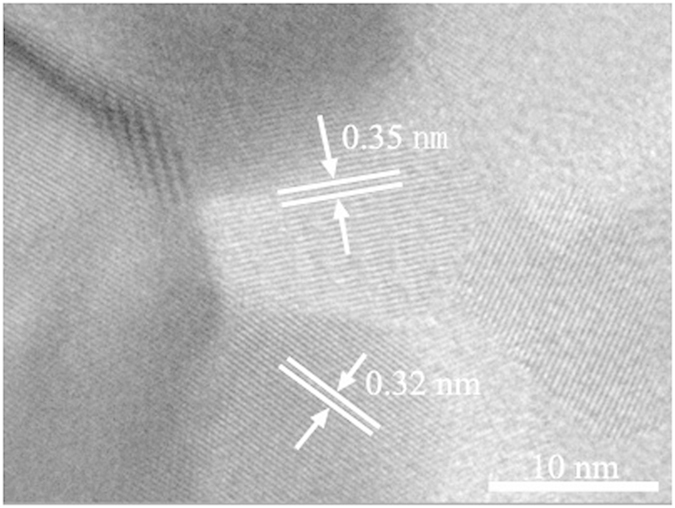
HRTEM image of the phase-junction TiO_2_ sample (W4).

In order to further demonstrate the existence of phase junction, the synthesized TiO_2_ samples were subjected to HRTEM imaging on one single particle. Figure 5 shows the HRTEM image of the sample W4, which shows one particle (Fig. 4). The lattice spacing of 0.32 nm, which corresponds to the (110) plane of the rutile lattice, could be clearly observed. In addition, the lattice spacing of neighbor nanoparticle was 0.35 nm, which corresponds to the (101) plane of the anatase lattice. Thus, the HRTEM observation confirms the existence of anatase-rutile phase junction.

### Photophysical properties of the TiO_2_ nanocomposites

**Figure 6:**
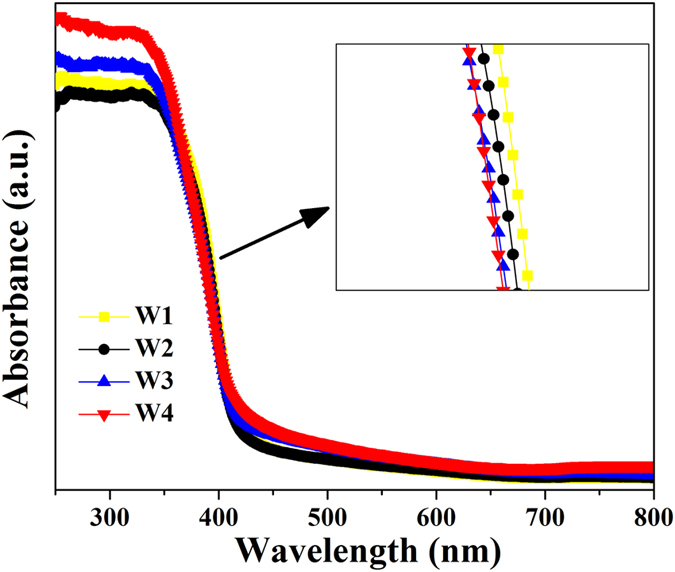
(**a**) UV-vis absorbance spectra of the phase-junction TiO_2_ samples and (**b**) PL spectra of anatase (W5) and phase-junction TiO_2_ (W4).

The optical absorption property of semiconductor materials, an important factor to evaluate their photocatalytic activity, is strongly related with their electronic band structures. The UV-vis absorbance spectra of the prepared TiO_2_ samples exhibit an absorption edge at about 420 nm (Fig. 6). Compared with W1-W3, W4 exhibited a slight blue shift. This result is reasonable as the less rutile phase decreases the contribution to the photon absorption range^40^. The order is consistent with the decreasing rutile content in the phase-junction TiO_2_. Furthermore, a successive increase in the absorption at λ < 400 nm as a function of decreasing rutile content could be observed. The UV light absorbance of the sample W4 with a rutile content (11.6%) exhibited the highest UV absorbance. The increased UV absorbance might play an important role in h^+^/e^−^ separation^41^. Thus, these results can be attributed to a charge separation, in which electrons transfer from rutile to anatase and holes move in the opposite direction^18^,^40^.

On the other hand, the phase-junction TiO_2_ exhibited a much lower photoluminescence (PL) intensity than the anatase TiO_2_ in the wavelength range of 300~700 nm (Supplementary Figure S4). Since PL is the emission of fluorescence originating from the recombination of photogenerated electron-hole pairs, the decreased PL intensity of the phase-junction TiO_2_ indicates a lower density of recombination centers and consequently higher availability of effective photogenerated carriers for catalytic reactions^42^. It is the reason why the photogenerated electrons and holes of rutile phase TiO_2_ are much less than those of the other samples. Such a decreased electron-hole recombination might be ascribed to an accelerated transfer of the photogenerated electrons from rutile phase to anatase phase of the phase-junction TiO_2_^43^.

### Photocatalytic activity of the TiO_2_ nanocomposites

**Figure 7:**
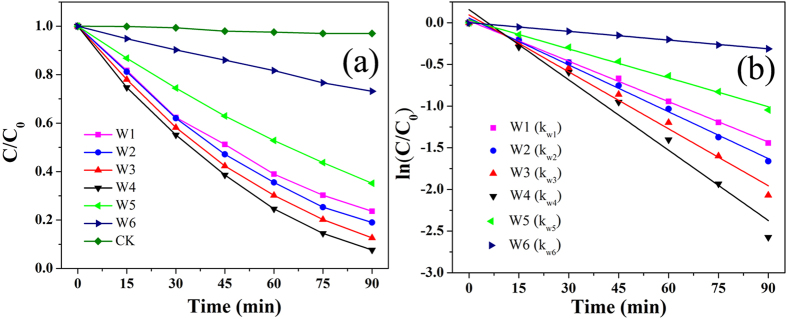
Photocatalytic degradation of RhB in aqueous solution under UV-vis irradiation in the presence of the phase-junction TiO_2_ photocatalys, anatase, and rutile, respectively. Here, C_0_ and C refer to the RhB concentrations at *t* = 0 and *t* = t, respectively.

Photocatalytic experiments were conducted to evaluate the photocatalytic activities of the prepared catalysts at various rutile to anatase ratios. Figure 7a shows the photocatalytic degradation of RhB by the TiO_2_ nanocomposites. The blank control (without photocatalyst) showed a stable concentration of RhB under UV-vis irradiation. By contrast, the presence of the TiO_2_ resulted in a rapid decrease in the RhB concentration. As shown in Fig. 6a, the photocatalytic capabilities of the phase-junction TiO_2_ nanocomposites were better than those of the anatase (W5) and rutile (W6). The highest photodegradation efficiency (92.4%) of RhB was achieved on the sample (W4) containing 11.6 wt% rutile after 90-min irradiation. The samples prepared with 51.8, 46.9, and 34.4 wt% rutile showed the total RhB photodegradation efficiencies of 76.4, 81.0, and 87.4%, respectively. For all the samples, the RhB photodegradation efficiency significantly increased with the decreasing amount of rutile to 11.6 wt%. This result indicates that both rutile to anatase ratio and surface phase junction play important roles in governing the recombination of photogenerated h^+^/e^−^ pairs for the phase-mixed TiO_2_^44^, and accordingly influencing its photocatalytic activity. From UV and visible Raman spectra, W4 with more anatase in bulk region and less rutile on surface, exposed more phase-junction on TiO_2_ surface, which is beneficial as the photogenerated electrons and holes could be trapped by oxygen and absorbed H_2_O to form active species^15^.

The RhB photodegradation process over time is illustrated in Fig. 7b, which follows the first-order kinetics. Thus, the apparent RhB degradation rate constant (*k*) could be calculated from the slope of the ln(*C*/*C*_*0*_) versus time fitting line, where *C* represents the RhB concentration. A comparison of the RhB degradation rate constants with different photocatalysts in Table 1 reveals that the fastest RhB degradation was achieved by the phase-junction TiO_2_. In addition, *k*_W4_ was larger than *k*_W1–3_ and *k*_W5,6_ in absolute value, implying that, to some extent, a lower proportion of rutile in the phase-junction TiO_2_ is favorable for strengthening its photocatalytic capability.

**Figure 8:**
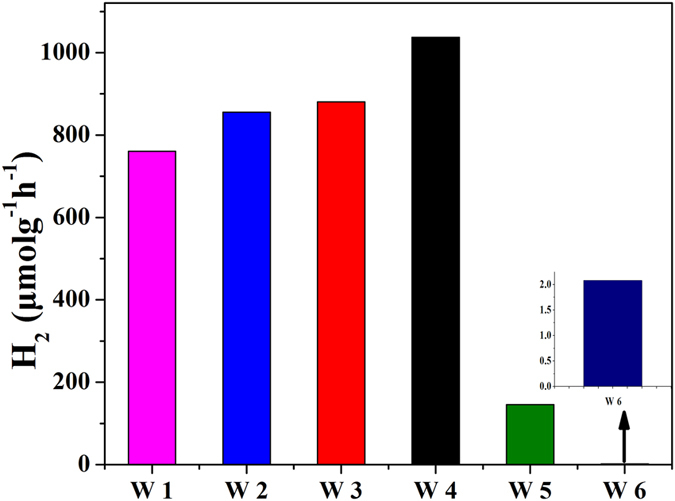
Time course of evolved H_2_ under UV-vis irradiation in the presence of the phase-junction TiO_2_ (W1-4), anatase (W5), and rutile (W6) catalysts, respectively.

The photocatalytic H_2_ evolution from water/methanol by the phase-junction TiO_2_ with different rutile to anatase ratios (deposited with Pt as cocatalyst) was investigated. The TiO_2_ samples were calcinated at the same temperature with different concentrations of precursor. Figure 8 shows that the H_2_ evolution rate changed with the rutile to anatase ratio and the H_2_ evolution rate of the phase-junction TiO_2_ was greater than those of the anatase (W5) and rutile (W6). Especially, the overall photocatalytic activity was more directly related to the surface region and the rutile content of the catalyst. The H_2_ evolution rate increased with the decreasing rutile content from W1 to W4. Meanwhile, the photocatalytic H_2_ evolution from water/methanol by the phase-junction TiO_2_ without Pt nanoparticles was investigated. As shown in Supplementary Figure S5, a small amount of H_2_ evolution at the beginning was attributed to the photogenerated-electrons that were utilized for forming the oxygen vacancies. After 1 h, the subsequent H_2_ evolution rate tended to be stable, exhibiting the similar tendency in the efficiency for the four phase-junction TiO_2_ nanoparticles (without Pt). Notably, the change of rutile to anatase ratio did not alter the surface phase and bulk region according to UV and visible Raman spectra. Thus, this result suggests that the surface-phase junction containing main anatase in bulk region and a little rutile on surface is more favorable for H_2_ production. For phase-junction TiO_2_ with more anatase in bulk region and less rutile, more H_2_ would be produced.

## Discussion

The above results demonstrate that the phase-junction TiO_2_ is responsible for the high photocatalytic activity. The formation of the surface phase junctions between the anatase and rutile nanoparticles enables effective interparticle electron transfer, which results in more efficient charge separation^15^,^45^,^46^. In addition, the two-phase mixed TiO_2_ with a small amount of rutile exhibited a higher photocatalytic activity. The photocatalytic activity of the samples decreased at a higher rutile content because the surface of the anatase TiO_2_ might be fully covered by the rutile nanoparticles, which reduced the amount of exposed anatase/rutile phase junction on the TiO_2_ surface.

**Figure 9:**
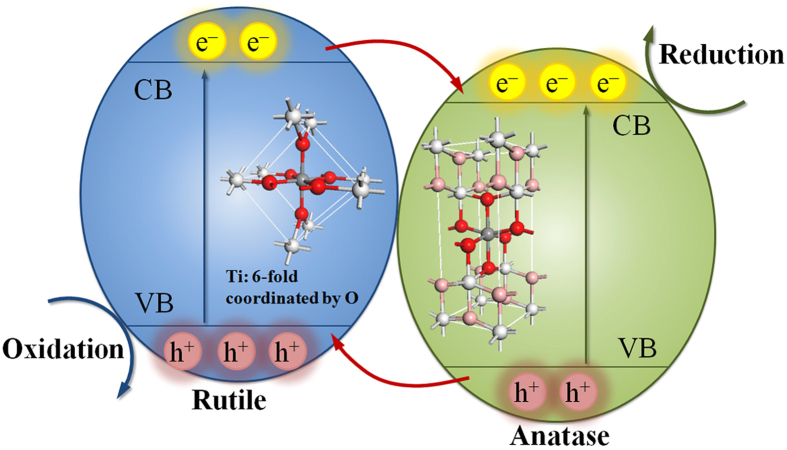
A proposed valence and conduction band alignment mechanism for the anatase/rutile interface. Red arrows indicate the flow of electrons (holes) in the conduction band (valence band). e^−^ and h^+^ represent electrons and holes, respectively.

On the basis of the above analysis, the mechanism for the photocatalysis of the phase-junction TiO_2_ under UV-vis irradiation is proposed (Fig. 9). The phase- junction samples allow electrons to flow from rutile to anatase, while the holes move in the opposite direction. Thus, the separation of photoexcited h^+^/e^−^ pairs is promoted^40^,^47^. The greater photoactivity of the TiO_2_ is mainly attributed to a synergistic effect between the two phases.

In the photocatalytic degradation of RhB, **·**OH radicals play a predominant role^46^. The excited electrons and transferred electrons on the surface of TiO_2_ can be trapped by oxygen to form **·**O_2_^−^, **·**HO_2_ and H_2_O_2_, then the **·**O_2_^−^ or electrons can further react with H_2_O_2_ to generate **·**OH. The photogenerated holes on the surface of TiO_2_ react with OH^−^ and H_2_O to produce **·**OH^48^. The **·**OH radical, as the main reactive species, can attack pollutant for degradation^49^. Consequently, not only an efficient consumption of the photo-induced h^+^/e^−^ pairs can be achieved, but also the h^+^/e^−^ recombination (fluorescence process) can be suppressed (Supplementary Figure S6a). The transfer of the photogenerated carriers (h^+^and e^−^) in the semiconductor photoexcitation pathways for the generation of reactive species is shown in Supplementary Figure S6b.

Likewise, in the photocatalytic H_2_ evolution, the excited electrons can be transferred to Pt nanoparticles of the Pt-loaded TiO_2_ catalysts. This would strengthen the interaction between Pt and TiO_2_ matrix as well as the interfacial electron transfer and h^+^/e^−^ separation, resulting in more efficient photocatalytic water splitting for H_2_ evolution^50^. Therefore, the phase-junction TiO_2_ has a higher UV-visible photocatalytic activity for both photooxidation (i.e., dye decomposition) and photoreduction (i.e., water splitting for H_2_ evolution). In such a TiO_2_ nanocomposite, the rutile to anatase ratio governs the efficiency of interfacial electron transfer and thus its photocatalytic activity.

## Conclusions

A simple but effective approach is developed to synthesize phase-junction TiO_2_ nanocomposites with a tunable ratio of rutile to anatase. In this approach, neither adjustors for tuning the ratio nor temperature adjustment is need to make phase transition. The experimental results indicate that the precusor concentration governs the synthesis of the phase-junction TiO_2_ nanocomposites through affecting the rutile content. The rutile content reduces with the decreasing Ti precusor concentration, while the hydrothermal time and temperature have no distinct effect on the rutile to anatase ratio. The photocatalytic activity of the TiO_2_ nanocomposites is significantly affected by the rutile to anatase ratio. A synergy between the two phases within a certain ratio range is observed. Compared with the other catalysts, W4 prepared by annealing the hydrothermal solution containing 0.1 g Ti and 40 ml HCl (6 mol/L), which has 11 wt% rutile content, exhibits the most excellent photocatalytic performance, because it reduces the rutile content in bulk region and makes rutile generate surface-phase junction, which promotes the charge separation. An appropriate ratio of rutile to anatase enables the formation of the surface-phase junctions in direct contact to facilitate the electron transfer between the two phases. This work not only offers a strategy to prepare unique nanocrystalline phase-junction TiO_2_, but also deepens our understanding about the properties of the phase-mixed TiO_2_ in photocatalysis, photoelectrochemical and photoelectric applications. Furthermore, such a phase-junction semiconductor may stand out as a promising candidate in environment remediation and the photoinduced splitting of water into hydrogen.

## Methods

### Rutile/anatase composite synthesis

All chemicals used in this work were of analytical-grade and used without further purification. The hydrothermal solution was prepared using a modified method from Guo *et al*^51^. Briefly, a 0.30 mm thick Ti foil was cleaned with acetone, alcohol and deionized water sequentially. The obtained Ti foils of different weights (0.6, 0.4, 0.2, 0.1, and 0.05 g) were then added into Teflon-lined stainless autoclaves with 50 mL of effective volume, and a mixture of water (20 mL) and concentrated hydrochloric acid (20 mL) was slowly added into each autoclave. Thereafter, the samples were autoclaved at 160 °C for 2 h and then naturally cooled under room temperature to obtain purple solutions without any precipitation. The ionic species in this purple solution is Ti^3+^ ion derived from Ti foil, as evidenced by the low temperature electron paramagnetic resonance (EPR) spectra (Supplementary Figure S7). The detailed measurement results of the Ti^3+^ concentration (Supplementary Figure S8) are described in Supplementary Information. Corresponding to four different weights of Ti foil, the obtained purple solutions with four different concentrations of Ti^3+^ were used in the subsequent synthesis.

Rutile/anatase-mixed TiO_2_ nanocomposites were prepared as follows: the as-prepared purple solution was annealed at different temperatures (300, 350, 400, 450, 500, 600, 700, and 800 °C (denoted as W6)) with a heating rate of 5 °C/min in air. The samples are cooled rapidly after reaching the above temperature.

The as-prepared solutions with different ratios of Ti foil to HCl were annealed at 500 °C for 2 h with a heating rate of 5 °C/min in air, and cooled to room temperature naturally. Thus, the samples with different weights of Ti foils were obtained, which were denoted as W1, W2, W3, W4, and W5 according to their ratios of Ti foil to HCl.

To prepare Pt/phase-junction TiO_2_ composites, the cocatalyst Pt (0.1 mg) was photodeposited on the rutile/anatase TiO_2_ catalyst (100 mg) *in situ* with H_2_PtCl_6_·6H_2_O as the precursor. Then, the Pt-loaded TiO_2_ samples were cleaned with water, and annealed at 450 °C for 0.5 h with a heating rate of 5 °C/min in air.

### Characterization

The low-temperature electron paramagnetic resonance (EPR) spectra of the obtained purple solution were recorded at 150 K to confirm the presence of Ti^3+^ ions (JES-FA200, JEOL Inc., Japan). Ti^3+^ concentration was determined with the method of redox titration by TIM865 titration manager (Radiometer Analytical, Villeurbanne, France). The crystal phase of the obtained samples were determined by X-ray diffraction (XRD) analysis using a Rigaku diffractometer (TTR-III, Rigaku Co., Japan), with a Cu Kα radiation source (λ = 1.541841 Å). The XRD scan speed was 8°/min, and the accelerating voltage and current were 40 kV and 200 mA, respectively. Visible and UV Raman spectra were recorded at ambient temperature using a Raman spectrometer (LabRamHR, HORIBA Scientific Inc., Japan) with an excitation wavelength of 514.5 nm generated by an Ar^+^ laser or 325 nm generated by a He-Cd laser. The Pt element in the phase-junction TiO_2_ samples after loading the Pt nanoparticles was analyzed by using inductively coupled plasma atomic emission spectrometry (ICP-AES) (Optima 7300 DV, PerkinElmer Co., USA). Morphology and structure of the samples were characterized by a transmission electron microscopy (TEM) (JEM2100, JEOL Inc., Japan). The high-resolution transmission electron microscopy (HRTEM) images were taken on an HRTEM (JEM-2010, JEOL Inc., Japan) at an acceleration voltage of 200 kV. The optical absorbance spectra of the samples were obtained using a UV–vis spectrophotometer (SOLID3700, Shimadzu Co., Japan). The PL measurement of the samples was carried out with a photoluminescence spectrophotometer (5301, Shimadzu Co., Japan).

### Photocatalytic activity evaluation

Photoactivities of the phase-junction TiO_2_ samples for RhB degradation and H_2_ evolution under UV-vis illumination were measured. Photocatalytic degradation of RhB was tested in a 50-mL beaker with a cold bath. In each beaker, 20 mg of the TiO_2_ catalyst was dispersed in a 30-mL RhB solution. Absorption spectrum of RhB was recorded using a UV-vis spectrophotometer (UV-2401PC, Shimadzu Co., Japan). Photocatalytic H_2_ evolution from methanol/water solution with the Pt-loaded TiO_2_ was investigated using a quartz reaction cell connected to a closed gas circulation and evacuation system. The light source was a 350 W (15 A) Xenon lamp (CHF-XM-350W, Beijing Trusttech. Co., China). The 0.1 wt % Pt-loaded catalyst (50 mg) was dispersed in an aqueous methanol solution (10 mL of CH_3_OH and 90 mL of H_2_O) with magnetic stirring. The H_2_ yield was quantified by a gas chromatograph (GC) (SP-6890, Lunan Co., China) equipped with a thermal conductivity detector and Ar gas as the carrier.

## Additional Information

**How to cite this article**: Wang, W.-K. *et al*. Self-induced synthesis of phase-junction TiO_2_ with a tailored rutile to anatase ratio below phase transition temperature. *Sci. Rep*. **6**, 20491; doi: 10.1038/srep20491 (2016).

## Supplementary Material

Supplementary Information

## Figures and Tables

**Table 1:** Rate Constants (*k*) and Linear Correlation Coefficient (*R* ^2^) of RhB Degradation with Different Rrutile Contents.

Catalyst	Rutile content (wt %)	*k*(min^−1^)	*R*^2^
W1	51.8	−0.016	0.998
W2	46.9	−0.018	0.996
W3	34.4	−0.022	0.987
W4	11.6	−0.028	0.973
W5	0	−0.012	0.994
W6	100	−0.004	0.998
