# Supplementary material for: Self-induced synthesis of phase-junction TiO2 with a tailored rutile to anatase ratio below phase transition temperature
Source: Sci Rep. 2016 Feb 11;6:20491. doi: 10.1038/srep20491 (PMC4750061; doi:10.1038/srep20491)
Supplement: Supplementary Information [file srep20491-s1.doc]

**Supplementary Information for**

**Self-induced synthesis of phase-junction TiO_2_ with** **a tailored rutile to anatase ratio below phase transition temperature**

Wei-Kang Wang, Jie-Jie Chen, Xing Zhang, Yu-Xi Huang, Wen-Wei Li, Han-Qing Yu^*^

Department of Chemistry, University of Science & Technology of China, Hefei, 230026, China

**Corresponding author:**

Prof. Han-Qing Yu, Fax: +86-551-63601592, E-mail: [hqyu@ustc.edu.cn](mailto:hqyu@ustc.edu.cn)

**Evidence for the Existence of Ti^3+^ Ion in the Purple Solution**

The purple solution without any precipitation could be obtained from the hydrothermal reactions of Ti foil and a mixture of water and concentrated hydrochloric acid in Teflon-lined stainless autoclaves. The existence of the Ti^3+^ ion in this purple solution was verified through the low temperature electron paramagnetic resonance (EPR) spectra (**Figure S7**). The signal observed at g= 1.97 of the EPR spectra is the paramagnetic characteristic of Ti^3+^ ion, thus confirming the formation of the Ti^3+^ ion from the Ti foil.

In addition, TIM865 titration manager was used to determine the concentration of Ti^3+^ ion in the purple solution. The principle of the redox titration is based on the following redox reaction:

3Ti^3+^ + Cr(VI) → 3Ti^4+^ + Cr(III) (1)

The consumption amount of Cr(VI) could be obtained in the process of redox titration (**Figure S8**a). And the concentration of Ti^3+^ ion can be calculated from the parameter of the isoelectric point. The mole amount of Ti^3+^ ion in purple solution equals with the one of Ti foil, indicating that only the Ti^3+^ ion as the Ti source exists in the solution.

Furthermore, the Ti^3+^ ion could be coordinated with H_2_O and Cl^–^ to form the substance of Ti(6H_2_O)Cl_3_ as the precursor for further application in the subsequent steps of the synthesis. The formation reactions are proposed as follows:

2Ti (foil) + 6H^+^ → 2Ti^3+^ + 3H_2_ (2)

Ti^3+^ + 6H_2_O + 3Cl^–^ → Ti(6H_2_O)Cl_3_ (3)

The composition of Ti(6H_2_O)Cl_3_ in the purple solution was verified by the XRD pattern (**Figure S8**b) of the substance from the purple solution after freeze-drying.


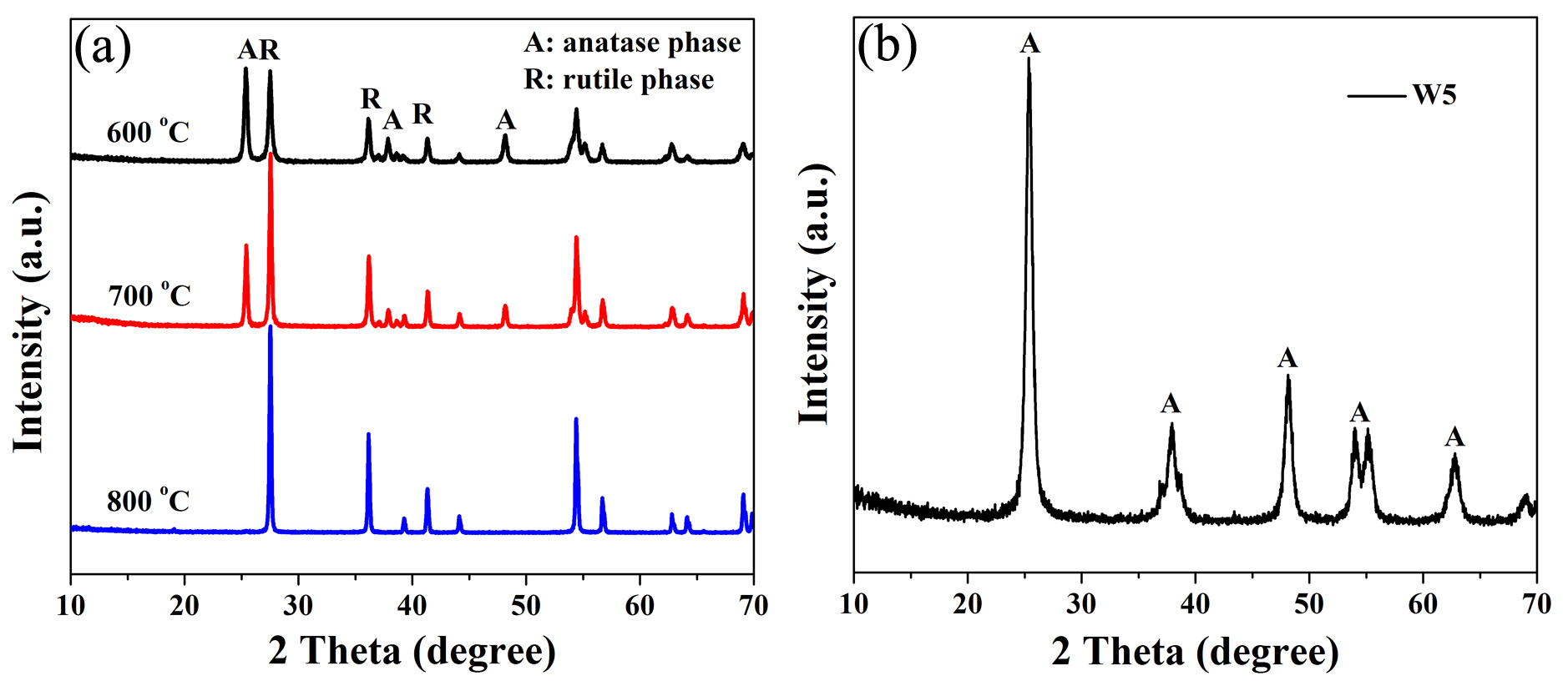


**Figure S1.** XRD pattern of (a) the phase-mixed TiO_2_ samples prepared at various annealing temperatures, and (b) the sample obtained with 0.05 g of Ti (W5)


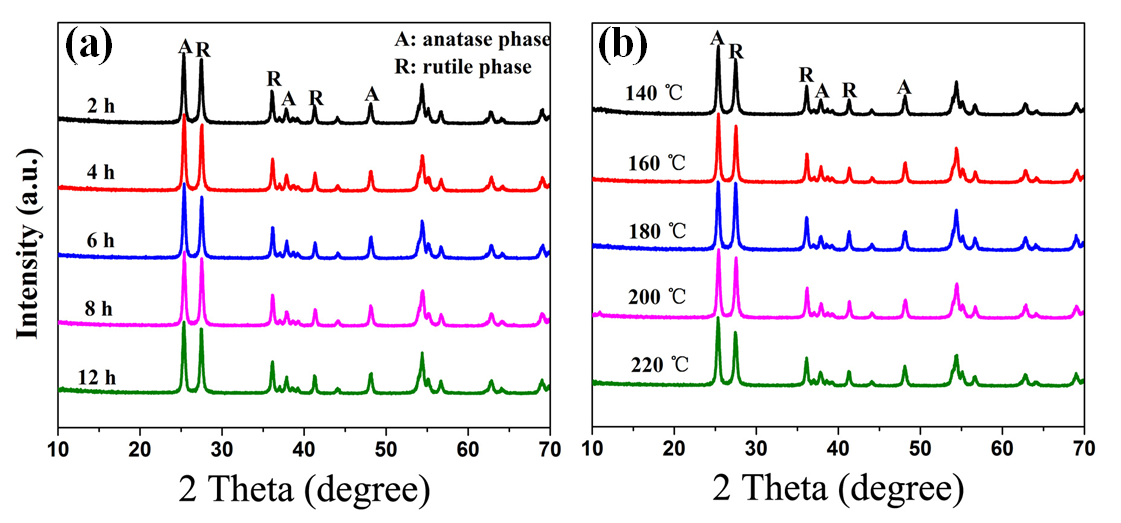


**Figure S2.** XRD pattern of the phase-mixed TiO_2_ samples prepared at various hydrothermal times (a) and different Ti precursor preparation temperatures (b), the rutile to anatase ratio of the nanocomposites was not substantially affected by the hydrothermal time or the Ti precursor preparation temperature


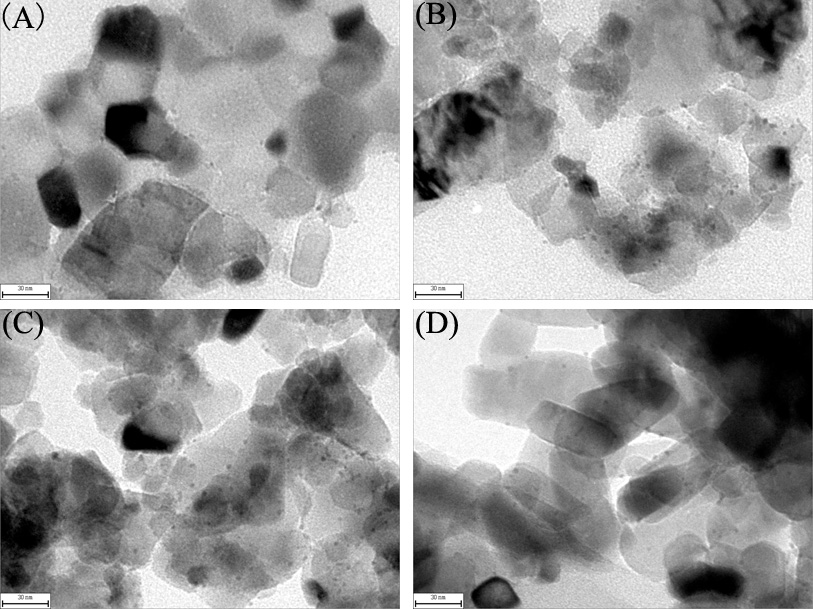


**Figure S3.** Typical TEM images of the phase-junction TiO_2_ sample W1 (a), W2 (b), W3 (c), and W4 (d) after loading Pt nanoparticles


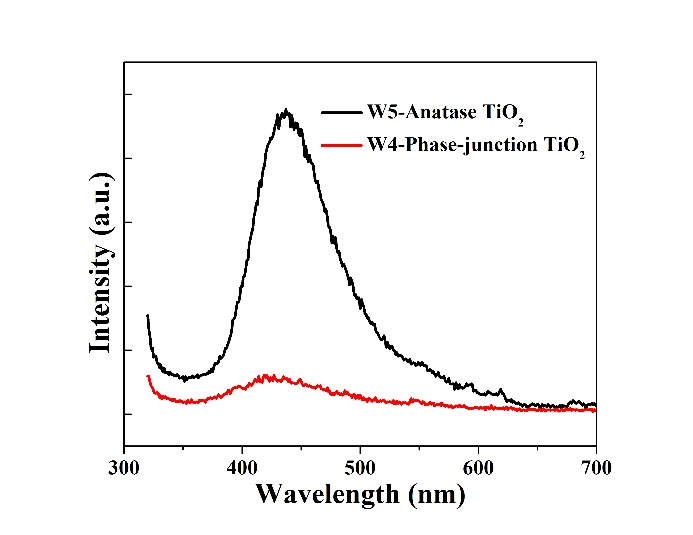


**Figure S4.** PL spectra of anatase (W5) and phase-junction TiO_2_ (W4)


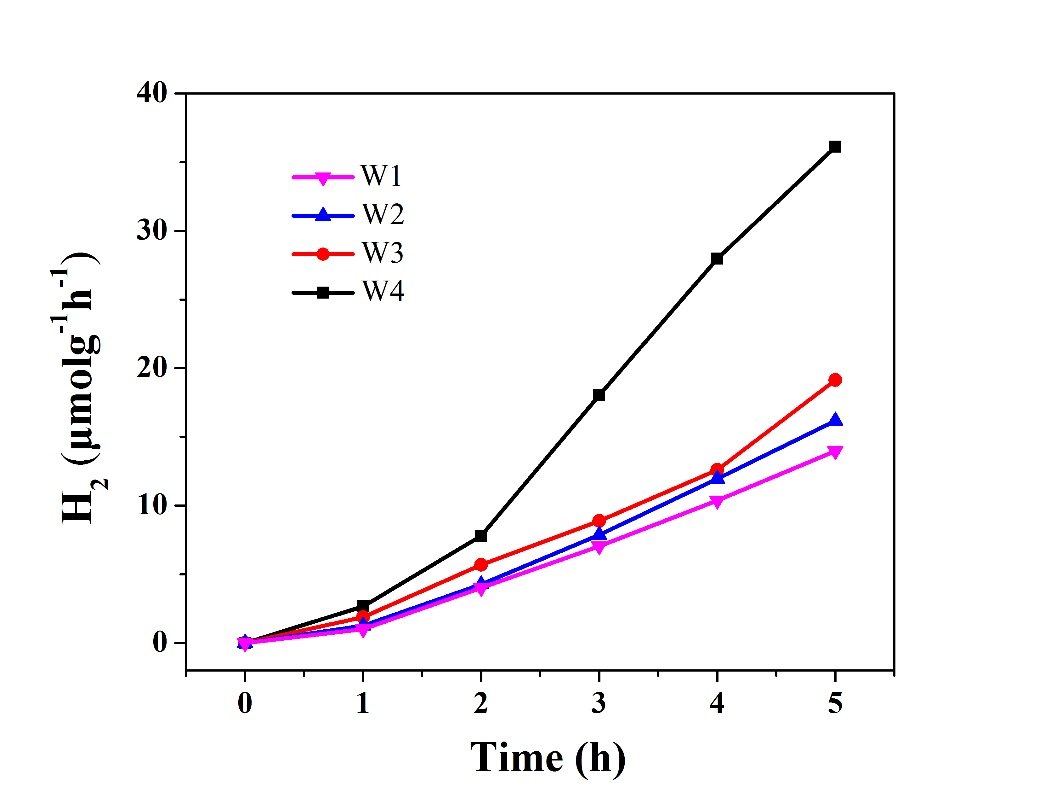


**Figure S5.** Time course of evolved H_2_ under UV-vis irradiation in the presence of the phase-junction TiO_2_ without Pt nanoparticles


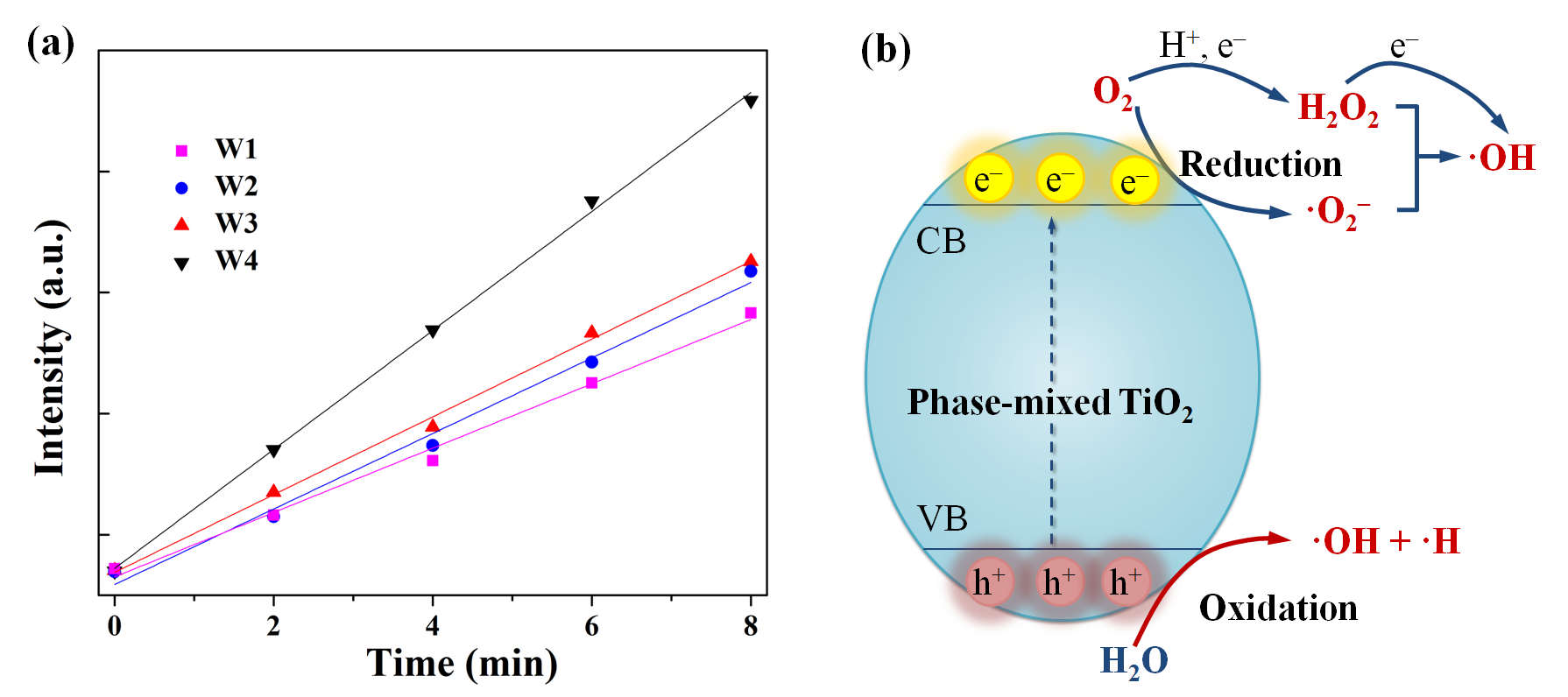


**Figure S6.** (a) Time dependence of the fluorescence intensity at 426 nm of all the phase-mixed TiO_2_ samples; and (b) formation processes of ·OH radicals on the conduction band and valence band through photogenerated electrons and holes, respectively


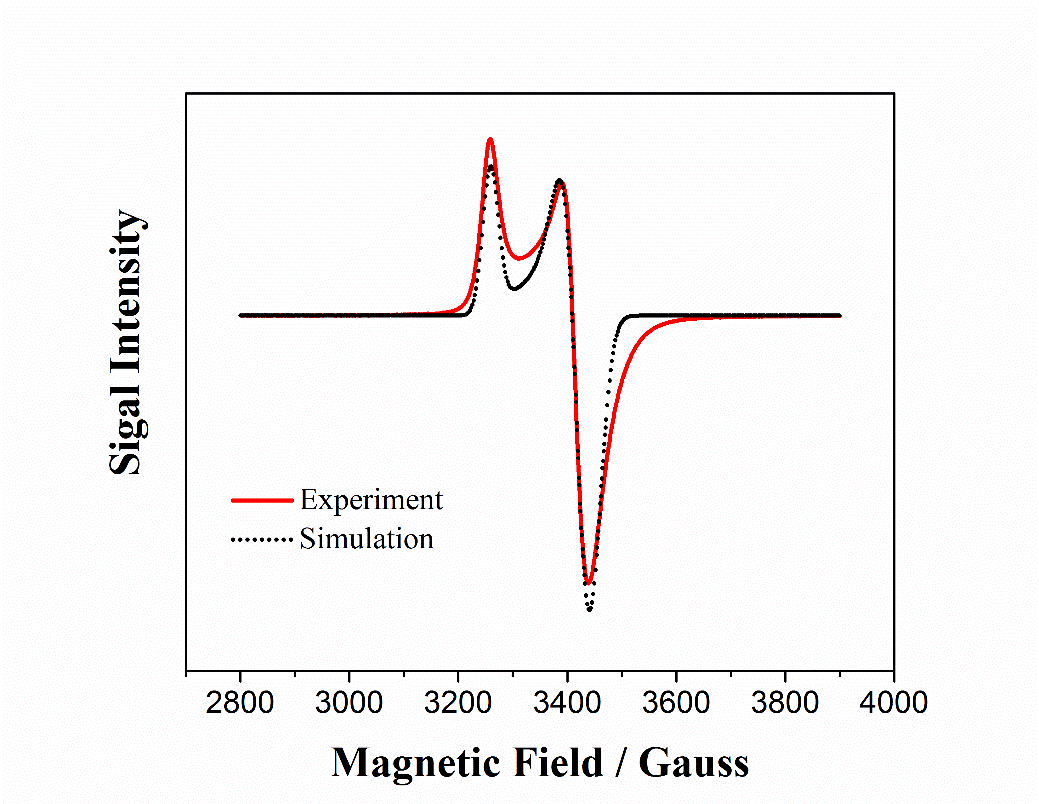


**Figure S7.** Low temperature EPR spectra of the obtained purple solution recorded at 150 K to confirm the presence of Ti^3+^ ions


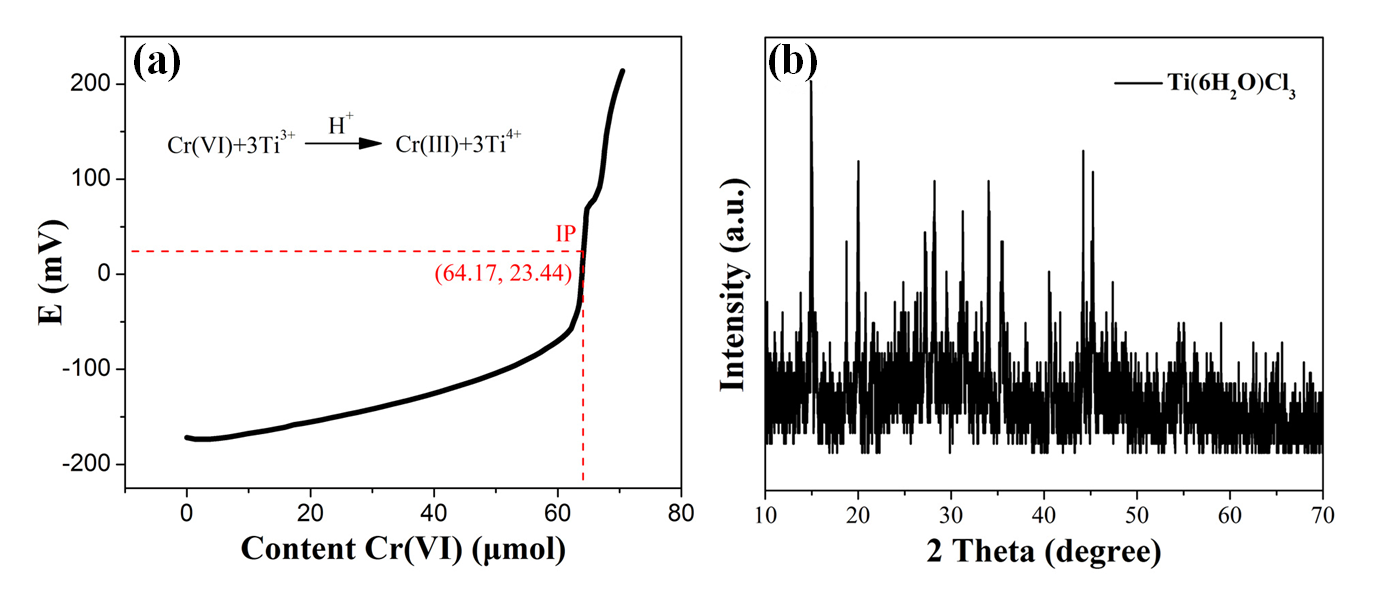


**Figure S8.** (a) Redox titration of Ti^3+^ concentration by using TIM865 titration manager; and (b) XRD pattern of the sample from the purple solution after freeze-drying, indicating that the sample presents the characteristic of Ti(6H_2_O)Cl_3_ crystal (JCPDS No. 17-343)
